# Carcass Characteristics and Primal Pork Cuts of Gilts, Boars, Immunocastrates and Barrows Using AutoFOM III Data of a Commercial Abattoir

**DOI:** 10.3390/ani10101912

**Published:** 2020-10-19

**Authors:** Kevin Kress, Jens Hartung, Johannes Jasny, Volker Stefanski, Ulrike Weiler

**Affiliations:** 1 Department of Behavioral Physiology of Livestock (460f), Institute of Animal Science, University of Hohenheim, Garbenstraße 17, 70599 Stuttgart, Germany; volker.stefanski@uni-hohenheim.de (V.S.); weiler@uni-hohenheim.de (U.W.); 2 Department of Biostatistics (340c), Institute of Crop Science, University of Hohenheim, Fruwirthstraße 23, 70593 Stuttgart, Germany; jens.hartung@uni-hohenheim.de; 3 Department of Agricultural Markets (420b), Institute of Agricultural Policy and Markets, University of Hohenheim, Schwerzstraße 46, 70593 Stuttgart, Germany; johannes.jasny@uni-hohenheim.de

**Keywords:** AutoFOM III, boars, carcass characteristics, entire male pigs, immunocastration, Improvac, primal pork cuts, surgical castration

## Abstract

**Simple Summary:**

Male piglets designated for pork production have been surgically castrated for centuries. The fattening of intact boars is more environmentally friendly due to anabolic effects, but entails a higher risk of aggressive and sexual behavior, and some boar carcasses may exhibit boar taint, which reduces pork quality. Immunocastration as a further alternative to surgical castration is considered as a reliable and animal welfare-friendly method, but currently still has a very small market share. A crucial factor for the period following surgical castration is that pork production, either with boars or immunocastrates, is competitive and produces valuable carcasses with regard to lean meat content and the respective amounts of primal pork cuts. In this study, we therefore evaluated AutoFOM III data from a commercial abattoir. The results show that carcasses from immunocastrates were of similar quality to those of barrows, with carcass characteristics that lay between the values of gilts and barrows. Boar carcasses are leaner in comparison to barrows, but produce the same amount of valuable pork per carcass. Boars and immunocastrates are therefore suitable for the processing industry and both techniques are able to compete with pork production with barrows in terms of carcass characteristics and primal pork cuts.

**Abstract:**

The surgical castration of male piglets as a routine procedure in modern pig production is facing increasing societal criticism. Pork production with boars and immunocastrates are available alternatives, but both have low market shares as it is so far uncertain how the carcass characteristics and primal pork cuts of boars and immunocastrates will be estimated in comparison to barrows and gilts. This article therefore evaluates the impact of sex group (gilts, boars, immunocastrates and barrows) on carcass characteristics and primal pork cuts using AutoFOM III data from a commercial abattoir. In our study, weekly slaughter data from a total of *n* = 36,994 pigs between 2018 and 2019 were analyzed. The results show that gilts had the highest amount of pork per carcass of all sex groups, whereas non-significant differences between boars, immunocastrates and barrows could be observed. Boars had the highest lean meat content, followed by gilts, immunocastrates and finally barrows with the lowest lean meat content. These results suggest that both immunocastration and pork production with boars are sustainable techniques that can replace pork production with barrows without affecting carcass quality.

## 1. Introduction

In Europe, the traditional practice of surgical castration of male piglets faces increasing societal criticism as it is painful and violates the animals’ physical integrity [1,2]. For this reason, European stakeholders voluntarily committed themselves in 2010 to end the surgical castration of male piglets by 2018 [3]. Despite this declaration, about 63% of male piglets in Europe are still surgically castrated within the first week of life [4]. Non-surgical alternatives (such as pork production with boars or immunocastrates) are available, but their practical acceptance is still limited. Boars may exhibit an unpleasant odor after entering puberty, the so-called boar taint, which reduces pork quality [5,6,7]. The market acceptance of immunocastration, on the other hand, although sustainable and above all animal friendly from a scientific point of view [8,9], has so far been impeded by a lack of practical experience in the pork production chain [9], and by market concerns as to whether the technique will be accepted within the industry [10,11].

Modern pork production is increasingly assessed based on sustainability, including economic, environmental, social and health aspects as well as animal welfare [12]. The advantages and disadvantages of pork production with boars or barrows with regard to sustainability are well-known and mainly linked to the presence of testicular steroids such as testosterone and estrogens with known anabolic properties [8], but also to the formation of androstenone, a testicular steroid contributing to boar taint [5]. Immunocastration as a further alternative to pork production with boars or barrows is an active immunization against the hypothalamic hormone GnRH [13] and consists of two consecutive vaccinations with Improvac^®^ (Zoetis Inc., Parsippany, NJ, USA) [14]. This technology has the potential to balance various advantages and disadvantages of pork production with boars or barrows, as from a physiological point of view, immunocastrates are boars until the second vaccination and barrows thereafter [13]. Depending on the requirements (process and product quality) of certain target markets, the corresponding advantages and disadvantages can be balanced by scheduling the timing of the second vaccination [9].

Regarding environmental and economic sustainability, it is important to analyze the amount of pork that can be produced depending on sex at a certain slaughter weight. In general, sexual dimorphism in the muscle growth of entire males in domestic animals is well-known, and boars develop more muscles in the shoulder and neck area than barrows or gilts due to testicular androgen secretion and the distribution of androgen receptors [15,16,17,18]. The meta-studies by Batorek et al. [19] and Nautrup et al. [20] compare the yield of various pork cuts of boars, immunocastrates and barrows. In both studies, the ham weight of immunocastrates was significantly higher than that of barrows, whereas belly and loin weights were similar. Nautrup et al. [20] described a higher shoulder weight for immunocastrates compared to barrows, whereas Batorek et al. [19] only detected a tendency for higher shoulder weight in immunocastrates. The weights of hams and shoulders were similar in immunocastrates compared to boars in both studies, whereas the belly weight of immunocastrates was significantly higher than that of boars [19,20]. Nautrup et al. [20] described a significantly higher loin weight in immunocastrates than in boars, whereas Batorek et al. [19] found no differences.

Commercial slaughterhouses, however, do not weigh each primal pork cut individually to calculate the economic value of a carcass, but estimate different primal pork cuts on the basis of the ultrasound technology AutoFOM III (Frontmatec, Kloding, Denmark) [21]. Based on the AutoFOM III results, the economic value of the carcass is determined and carcasses are then assigned to different sales channels [22]. The estimation formulas of AutoFOM III were based on the results of trials with barrows and gilts, for which the exact weight of individual primal pork cuts was determined after standardized dissection. These trials did not include data from boars and immunocastrates [23]. Thus, it remains to be clarified how the estimation of pork cuts of boars and immunocastrates differ from those of barrows or gilts. Our study therefore sets out to provide a comparison of carcass characteristics and different primal pork cuts based on AutoFOM III data between gilts, boars, immunocastrates and barrows.

## 2. Materials and Methods

### 2.1. Animals

Slaughter data from a total of 36,994 pigs originating from 11 farms in Baden-Württemberg (south-west of Germany) were evaluated to compare the carcass characteristics and weight of primal pork cuts from gilts (G, *n* = 15,242), boars (B, *n* = 16,949), immunocastrates (IC, *n* = 2822) and barrows (BA, *n* = 1981). The animals were fattened and slaughtered as part of the regional meat program “Gutfleisch” of EDEKA Südwest Fleisch GmbH (Rheinstetten, Germany). This meat program imposes special requirements on participating farms. Thus, housing conditions differ from the legal minimum requirements, as the animals have 10% more space (see [24]) and access to organic manipulable material is provided. The genotype of pigs used for pork production may vary as different breeds are used as dams depending on the farm, but the Piétrain genotype (German Piétrain INODORUS 2.0, German Genetic, Stuttgart, Germany) of the sire is mandatory (see Table 1). Immunocastrate (IC) pigs should be vaccinated twice with Improvac^®^ at a live weight of approximately 30 (first vaccination) and 80 kg (second vaccination, 4–6 weeks before slaughter). All animals were slaughtered at a regional slaughterhouse (Vion Crailsheim GmbH, Crailsheim, Germany). At this facility, the slaughter of boars and immunocastrates was restricted to one day per week (Tuesday), so the dataset includes only pigs slaughtered on Tuesdays during our 18-month observation period (January 2018 to June 2019). At slaughter, the hot carcass weight of each pig was recorded, and the lean meat percentage and primal pork cuts were estimated using AutoFOM III (Frontmatec, Kloding, Denmark).

### 2.2. Carcass Characteristics and Primal Pork Cuts

Each carcass was classified according to the German grading system for pig carcasses (SEUROP), based on its estimated lean meat percentage [25]. The German pricing system for pig carcasses rewards lean carcasses, and such carcasses are rated S [22,26]. The lean meat percentage was estimated using a fully automatic classification system for pig carcasses (AutoFOM III) licensed for the German rating system [25]. Fat and muscle thickness were measured along the carcass with 16 ultrasound probes, including 3200 individual measurements. Based on the carcass weight and said measurements, the weight of shoulder, ham, loin, belly and the overall lean meat percentage relevant for classification were estimated according to predetermined formulas [21,25].

### 2.3. Statistical Analysis

The dataset considered animals with a hot carcass weight between 50 and 120 kg, as carcasses with weights above or below these thresholds are not covered by the German grading system. As a consequence, 54 out of 36,994 observations were discarded prior to analysis. Data were analyzed using the following linear mixed model

(1) $$y_{ijklm}=\mu +\beta x_{ijklm}+\varphi_{j}+f_{jl}+\tau_{i}+d_{k}+{\left(\varphi \tau \right)}_{ij}+{\left(f\tau \right)}_{ijl}+e_{ijklm},$$

μ is the intercept and β is the slope for the covariate hot carcass weight ($x_{ijklm}$). $\varphi_{j}$ is the fixed effect of the *l*th dam line, $f_{jl}$ is the random effect of the *j*th farm within dam line *l*. $\tau_{i}$ is the fixed effect of the *i*th sex group and $d_{k}$ is the random effect of the *k*th slaughtering date. ${\left(\varphi \tau \right)}_{ij}$ and ${\left(f\tau \right)}_{ijl}$ are the random dam line-specific and farm-specific deviations from the general sex group effect. $e_{ijklm}$ is the error of observation $y_{ijklm}$ with *m* being an index for the pig. Equation (1) is modified for the analysis of hot carcass weight by removing the covariate. For all traits, residuals were graphically checked to fulfill normal distribution and homogeneous variance. In case of significant F-tests, a Fishers LSD test for the corresponding significant effects was performed [27]. Additionally, best linear unbiased estimators (BLUPs) were estimated for the farm effect. BLUPs were adjusted for the average hot carcass weight. Furthermore, the general mean was added so that BLUPs from farms have the same average as means for other effects.

In order to compare, the proportion of pigs in each sex group being graded in the German classification system were checked via an RxC table.

All analyses were performed using the SAS software Version 9.4.

## 3. Results

A mixed linear model analysis indicated that the dam line had an effect on hot carcass weight only. The line Danzucht showed significantly higher hot carcass weights compared to all other dam lines (99.52 vs. 91.10–94.17 kg). It is interesting to note that the dam line was significant for several traits if the hot carcass weight was not included in the analysis (results not shown). After including the hot carcass weight as covariable, the dam line was non-significant for all traits. Sex group is significant for all traits except for belly lean meat content (%), loin with bones (kg), lean and boneless loin (kg) and belly (kg) (Table 2). In the following section, we highlight the differences between the sex groups.

### 3.1. Impact of Sex Group on Carcass Characteristics and Primal Pork Cuts

Table 2 illustrates the differences between gilts, boars, immunocastrates and barrows with regard to various carcass characteristics and primal pork cuts. The hot carcass weight of immunocastrates was slightly higher compared to other sex groups, but differences between all sex groups were non-significant (*p* = 0.2804). In none of the other traits is a significant difference between barrows and immunocastrates evident. While differences between barrows and gilts or boars were significant for most traits, values for immunocastrates were generally intermediate, with either significant or non-significant differences compared to gilts or boars.

Sex groups differed significantly in lean meat percentage (*p* = 0.0003). Boars reached the highest lean meat content, followed by gilts, immunocastrates and finally by barrows with the lowest lean meat content. The differences between barrows, boars and gilts were significant (*p* = 0.0003 and *p* < 0.0001, respectively). A different scenario emerged regarding the belly lean meat content. Here, no differences between sex groups were significant (*p* = 0.0813). As with lean meat percentage, differences between sex groups were also significant with regard to fat thickness (*p* = 0.0005). Barrows had a higher fat thickness than gilts and boars (G vs. BA, *p* = 0.0005, B vs. BA, *p* < 0.0001), whereas non-significant differences existed between gilts and boars. The fat thickness of immunocastrates was again intermediate.

Muscle thickness differed significantly between sex groups (*p* = 0.0008). Gilts had the highest muscle thickness (G vs. B, *p* = 0.0002; G vs. IC, *p* = 0.0084; G vs. BA, *p* = 0.0225), followed by barrows, then boars with the lowest muscle thickness (B vs. BA, *p* = 0.0049). Immunocastrates showed non-significant differences in muscle thickness compared to boars or barrows (B vs. IC, *p* = 0.2499; IC vs. BA, *p* = 0.1940).

The primal pork cuts estimated by AutoFOM III also revealed a significant impact of sex groups (*p* < 0.0001) on all cuts except for the two loin weights and the belly weight. Loin weight with and without bones was 170–200 g lower in barrows compared to gilts, but did not differ significantly from each other.

Differences between sex groups were significant with regard to ham weight with bones and lean and boneless ham weight (*p* = 0.0010; *p* = 0.0007, respectively). Gilts showed the highest ham weight with bones compared to all other sex groups (G vs. B, *p* = 0.0011; G vs. IC, *p* = 0.0052; G vs. BA, *p* = 0.0285). Gilts also had significantly higher lean and boneless ham weight values than boars (*p* = 0.0006), immunocastrates (*p* = 0.0459) and barrows (*p* = 0.0003). For both ham weights, a non-significant difference between barrows and immunocastrates was obvious. Boars had a 70–400 g lower ham weight with bones compared to all other sex groups, with non-significant differences to immunocastrates (B vs. IC, *p* = 0.4628; B vs. BA, *p* = 0.0129). With regard to lean and boneless ham weight, boars did not significantly differ compared to immunocastrates and barrows.

The impact of sex groups on the shoulder weight with bones was also significant (*p* = 0.0179). Similar to ham weight with bones, there was no statistically significant difference between shoulder weight with bones between boars, immunocastrates and barrows. Gilts showed a higher shoulder weight with bones than boars (*p* = 0.0400) and barrows (*p* = 0.0025), whereas non-significant differences existed between gilts and immunocastrates (*p* = 0.2394). Boars and barrows did not differ statistically in their shoulder weights with bones. Sex groups differed significantly in their lean and boneless shoulder weights (*p* = 0.0067). The lean and boneless shoulder weight was higher in gilts and boars compared to barrows (G vs. BA, *p* =0.0007; B vs. BA, *p* = 0.0280). Immunocastrates were again intermediate with non-significant differences to the other sex groups.

Immunocastrates had the highest belly weight of all sex groups (+ 20–230 g), followed by boars, barrows and gilts. Differences between sex groups were non-significant (*p* = 0.0868).

**Figure 1:**
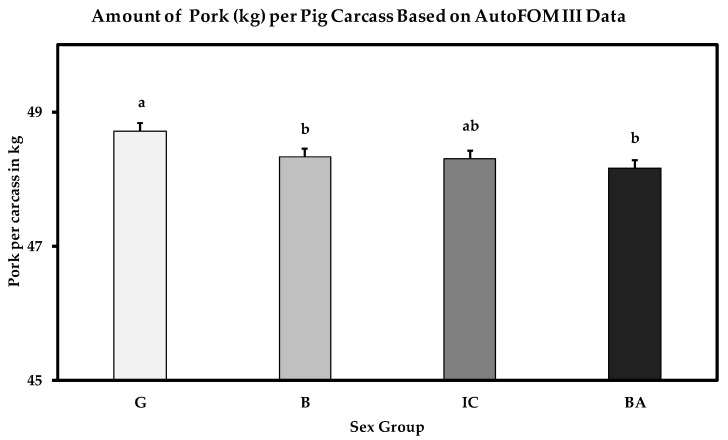
Amount of pork per carcass in kilograms from gilts (G; *n* = 15,226), boars (B; *n* = 16,922), immunocastrates (IC; *n* = 2815) and barrows (BA; *n* = 1977) based on AutoFOM III estimations (LSM ± SE). The amount of valuable pork (kg) between different sex groups with at least one identical letter shows no significant difference between them (*p* < 0.05).

The overall sum of valuable pork for human consumption (taking into account lean and boneless primal pork cuts and belly weight) differed between sex groups (*p* = 0.0015), with gilts reaching up to 48.71 ± 0.15 kg/carcass compared to boars with 48.33 ± 0.16 kg/carcass, immunocastrates with 48.30 ± 0.26 kg/carcass and barrows with 48.16 ± 0.15 kg/carcass (Figure 1). There were no statistically significant differences in the sum of valuable pork per carcass in immunocastrates compared to the other sex groups (G vs. IC, *p* = 0.1063; B vs. IC, *p* = 0.9106; IC vs. BA, *p* = 0.5786), or between boars and barrows (B vs. BA, *p* = 0.2054). Gilts produced significantly more valuable meat than boars and barrows (G vs. B, *p* = 0.0070; G vs. BA, *p* = 0.0002).

### 3.2. Impact of Sex Group on Carcass Classification (SEUROP)

As described in Section 2.2, the German pricing system for pig carcasses rewards lean carcasses. Thus, the proportion of a sex group within the highest quality grade (S) is a decisive factor for monetary compensation. The assignment of carcasses to quality grades of pig carcasses (SEUROP) varies between sex groups (*p* < 0.0001). Proportions are given in Table 3. Boars are most often classified as S (89.3% of all carcasses), followed by immunocastrates with 86.5% and gilts with 85.3%. In contrast, only 47.9% of barrows are classified as S and 42.1% as the second most valuable category E. In the other sex groups, the proportion of carcasses classified as E is between 10.5 and 14.3%. It is interesting to note that 9.0% of barrows are in the third grading, class U, whereas below 1% of the carcasses of all other sex groups were in class U. Gilts, boars and immunocastrates had no carcasses in grading class R, whereas 1% of barrow carcasses received an R. No carcasses were classified as O or P in any sex group.

### 3.3. Impact of Farms on Carcass Characteristics and Primal Pork Cuts

The BLUPs (best linear unbiased estimators) of the 11 farms are presented in Table 4 in order to determine the impact of farm on carcass characteristics and estimated primal pork cuts using AutoFOM III data. Farm B and the two farms, J and K, which are the only farms that fatten immunocastrates, have the lowest hot carcass weights (kg) of all farms. Farms with a lower hot carcass weight (kg) also have a higher proportion of lean meat content (%). With the exception of farm K, these farms do not necessarily produce a smaller amount of pork per carcass (kg). Farm B and J produced the highest amounts of pork per carcass (kg) of all farms despite having relatively low hot carcass weights (kg). This is reflected particularly in the lean and boneless loin, ham and shoulder weight (kg), but not in the belly weight (kg).

## 4. Discussion

Traditionally, male piglets designated for pork production are surgically castrated within the first week of life to prevent boar taint and to avoid male-specific aggressive and sexual behavior [1]. With regard to the current debate about the welfare of male piglets, which is negatively affected by surgical castration without pain relief, both pork production with boars and immunocastrates are immediately available as alternatives for the pork chain [8]. Due to sexual dimorphism, we know that intact boars produce leaner carcasses than gilts or barrows, while at the same time have a more pronounced shoulder and neck area [15,16,17]. In practice, the carcass parameters and primal pork cuts are estimated on the basis of AutoFOM III data and are not weighed individually for each carcass [21]. Since the AutoFOM III estimation is based on mathematical estimation formulas that were developed on the basis of dissection trials with gilts and barrows [23], it has so far been unclear how the carcass characteristics and primal pork cuts of boars and immunocastrates are estimated. The aim of this study was therefore to analyze the differences in carcass characteristics and primal pork cuts in gilts, boars, immunocastrates and barrows based on AutoFOM III data, and thus to indirectly determine the economic value of the carcasses.

The results of our dataset show non-significant differences in hot carcass weight between sex groups, with immunocastrates showing the highest values and boars the lowest. The meta-analysis by Nautrup et al. [20] shows a lower slaughter weight for boars compared to immunocastrates and barrows. Since the exact age of the animals in this study is not known, it is possible that boars were slaughtered at an earlier age than the other sex groups, as Germany uses a special pricing system for boars in which their optimal hot carcass weight is below that of gilts, immunocastrates and barrows [26]. On the other hand, a lower hot carcass weight of boars at a fixed age could also be due to the fact that intact boars with a Piétrain background have a lower feed intake and therefore grow more slowly despite having better feed utilization [28]. The slight but non-significant higher hot carcass weight of immunocastrates compared to barrows contrasts with the results of the studies by Pauly et al. [29] and Nautrup et al. [20], in which barrows had a higher hot carcass weight. These studies, however, refer to standardized trials with fixed ages at slaughter. It is well known that the growth performance of immunocastrates increases significantly after the second vaccination [19,30,31], as immunocastrates consume significantly more feed after the second vaccination [32]. For this reason, the timing of the second vaccination has a decisive impact on growth performance and thus indirectly on the final hot carcass weight of immunocastrates with a fixed fattening period. The non-significant lower hot carcass weight of gilts results from an overall lower growth performance in gilts compared to barrows [33,34] or immunocastrates [30], since the feed intake of barrows and immunocastrates is increased compared to gilts [30,33,34].

This paper for the first time evaluates AutoFOM III slaughter data from different sex groups (gilts, boars, immunocastrates and barrows) originating from different farms that were fattened under practical on-farm conditions. The lean meat content was highest in boars, followed by gilts, immunocastrates and lowest in barrows. Similar results were also obtained in a standardized study by Tanghe et al. [35] and in several dissection trials [19,20,36], and can be explained by a better protein deposition in intact boars and gilts due to the presence of androgens and estrogens [6,37]. In total, the highest amount of pork per carcass as estimated by AutoFOM III can be produced by gilts with non-significant differences to immunocastrates, whereas gilts produce more pork per carcass than boars and barrows. A meta-study by Nautrup et al. [20] revealed that the highest amount of valuable pork is produced by immunocastrates compared to barrows and boars. This was also confirmed in the meta-study by Batorek et al. [19] for shoulder and ham weights, whereas the belly weight of barrows was higher than that of immunocastrates. Our study showed that immunocastration leads to statistically non-significant differences of pork per carcass compared to boars and barrows, whereas carcasses from boars show slightly higher amounts of pork per carcass than immunocastrates and barrows. Thus, our results showed that switching to pork production with immunocastrates or boars would not be detrimental to overall pig production and could certainly compete with current products on the pork market.

The influence of immunocastration on the sustainability of pork production is further strengthened by other factors such as better feed conversion in immunocastrates than in barrows, as well as fewer boar-tainted carcasses and less aggressive behavior compared to boars [9]. The lower shoulder weight of boars compared to gilts contradicts the assumptions drawn from sexual dimorphism [15,16,17] and the results of other meta-studies [19,20], as well as a dissection trial by Bauer and Judas [36], in which boars had the highest shoulder weight compared to gilts and barrows. Immunocastrates, on the other hand, show intermediate shoulder weights in this study, with non-significant differences to boars and barrows. These results are also supported by the above-mentioned meta-studies. In these studies, immunocastrates showed the highest shoulder weight, but the differences between sex groups were also non-significant [19,20] since boars and immunocastrates are physiologically identical up to a few weeks before slaughter [13,38]. In our study, boars have the lowest ham weight with bones and their lean and boneless ham weight is also lower compared to gilts. This result contradicts the study by Gispert et al. [39], which found that ham proportions in boars in relation to carcass weight were significantly higher than in gilts, immunocastrates and barrows, and thus led to an overall higher ham weight. One hypothesis to explain the lower shoulder and ham weights in boars compared to gilts is that AutoFOM III imprecisely estimates the shoulder weight and possibly other primal pork cuts from intact boars. On the other hand, the ham weight with the bones of boars is the lowest in our study compared to all other sex groups, something which has also been found in a precise dissection trial with similar breeds as those used for our study [36]. Age also has an impact on the weight of the primal pork cuts. Due to allometric growth we know that both shoulder and ham weight increase along with an increasing hot carcass weight, but the relative growth of these muscle parts decreases compared to other pork cuts and tissues (such as fat, for example) [15]. The exact age of the animals in this study is not known, but we know the animals’ hot carcass weight, which is therefore included in our statistical model. The hot carcass weight does not differ significantly between the sex groups. As the genotype of the sire is Piétrain in all animals, it is rather unlikely that the intact boars were slaughtered at an earlier age than the other sex groups and thus have lower shoulder and ham weights than gilts. As mentioned above, it is well known that feeding behavior in intact boars is reduced, especially in the Piétrain genotype [40] and that the growth performance of boars is therefore lower than in the other sex groups [28]. In consequence, this would rather indicate that intact boars in our study might be somewhat older than the other sex groups, as their lower growth rates might have required more time to reach their final hot carcass weight. As mentioned above, however, the exact age of the animals in this study is not known.

Since the AutoFOM III data are estimates based on mathematical formulas developed on the basis of dissection trials with gilts and barrows [21,23], it is unclear how precisely these formulas also estimate the carcass characteristics and primal pork cuts of boars and immunocastrates. A validation study by Bauer and Judas [41], based on dissection trials with boars, shows that the AutoFOM III values for boars are less precise than those for barrows and gilts, but still within a tolerable range (as for rare breeds in gilts or barrows) and thus do not need to be adjusted. This could therefore explain the unexpected lower AutoFOM III shoulder weight of boars in this study. However, since the proportions of boars and immunocastrates within the pork market have increased in the past years [4,9,42], a more detailed dissection trial and validation study should be conducted for boars and immunocastrates. If necessary, the AutoFOM III estimation formula must be adapted or separate estimation formulas must be developed for each sex group in order to ensure fair pricing conditions for all sex groups and to avoid market distortions.

Our study also shows a higher variability of lean meat content in barrows and thus heterogeneous carcass qualities compared to the other sex groups. Heterogeneous carcass qualities are undesirable for both slaughterhouses and meat-processing facilities, as this results in a higher sorting effort [43]. Pork production with boars and immunocastrates thus minimizes the risk of heterogeneous carcass qualities and maximizes the profits, as it is less expensive to sort carcasses in order to produce uniform pork quality.

This study includes fixed effects for the genetic influence of the dam line and thus allows for random farm-specific deviations from these effects in the statistical model. As described in the review by Olsson and Pickova [44], it is well known that management (housing conditions, age, genetics, sex and feeding) can influence various pork quality parameters such as water holding capacity, fat content, tenderness, color and aroma. We also know that stress and intensive housing conditions can have a negative impact on growth performance and hot carcass weight in pigs [31,45]. An experiment with organic housing conditions and organic feed showed that the feed had no influence on the lean meat content of pig carcasses, but the housing conditions did. Animals that were fattened under organic housing conditions had a lower lean meat content than animals that were fattened under conventional conditions [46]. A recent study by Kress et al. [31] also highlighted that animals housed under stressful conditions due to repeated social mixing had a higher proportion of lean meat than animals in the standard and enriched groups [47], which could be attributed to increased lipolysis caused by stress [48]. An increasing animal/feeding-place ratio can also lead to more social stress in pigs and thus to a higher proportion of lean meat [49]. A study by Rehfeldt et al. [50] also showed that the birth weight of piglets has a significant impact on carcass characteristics. Animals with a high birth weight have a significantly higher hot carcass weight than animals with a low birth weight. In this study it was also shown that animals with a high birth weight also had larger ham areas than animals with a lower birth weight. It can therefore be concluded that birth weight has an indirect impact on primal pork cuts.

## 5. Conclusions

This study shows that, based on AutoFOM III data originating from 11 farms in Germany, boars as well as immunocastrates produce similar amounts of valuable pork per carcass for human consumption as barrows. Carcass characteristics and the weights of primal pork cuts do not differ significantly between immunocastrates and barrows. With regard to carcass characteristics such as lean meat content, values for immunocastrates are intermediate to those of gilts and barrows, whereas boars showed the highest lean meat content of all sex groups.

The extent to which the AutoFOM III estimation formulas provide precise data for boars and immunocastrates is unclear and must be further examined in a detailed dissection trial. Boars, in particular, may be misevaluated due to an increased proportion of lean meat and a different carcass composition due to sexual dimorphism. The variability of carcass qualities is lower and more homogenous in boars and immunocastrates compared to barrows. Both pork production with boars and with immunocastrates therefore offer the industry opportunities to produce carcasses with a constant quality and, at the same time, with non-significant differences in the amounts of pork per carcass for human consumption.

## Figures and Tables

**Table 1:** Dam line and number of pigs per sex group (gilts (G), boars (B), immunocastrates (IC) and barrows (BA)) from 11 farms in Baden-Württemberg (Germany) slaughtered on Tuesdays from January 2018 to June 2019 at the slaughterhouse of Vion Crailsheim GmbH (Germany) at a hot carcass weight between 50 and 120 kg.

Farm	Dam Line	G (*n*)	B (*n*)	IC (*n*)	BA (*n*)	∑
A	Danzucht	479	420	0	95	**994**
B	BW-Hybrid	1484	1570	0	114	**3165**
C	BW-Hybrid	1952	3816	0	84	**5843**
D	Large White x Danish Landrace	1402	2527	0	76	**4005**
E	Large White x Danish Landrace	645	1698	0	36	**2379**
F	Large White x Landrace	3324	3227	0	93	**6644**
G	Danzucht	677	703	0	90	**1470**
H	BW-Hybrid	1006	796	0	498	**2300**
I	Danish Landrace x Hybrid-Line	1184	2171	0	697	**4052**
J	Danzucht	2562	0	2367	58	**4987**
K	BW-Hybrid	517	0	448	136	**1101**
**∑**		**15,226**	**16,922**	**2815**	**1977**	**36,940**

The sum per farm or sex group is presented in bold letters.

**Table 2:** Carcass characteristics and estimated primal pork cuts using AutoFOM III data (LSM ± SE) of gilts (G), boars (B), immunocastrates (IC) and barrows (BA).

	Sex Group				
Parameter	G (*n* = 15,226)	B (*n* = 16,922)	IC (*n* = 2815)	BA (*n* = 1977)	*p*-Value
Hot carcass weight (kg)	94.22 ± 0.61	93.72 ± 0.62	94.94 ± 0.99	94.61 ± 0.35	*0.2804*
Lean meat content (%)	60.72 ± 0.55 ^a^	61.21 ± 0.57 ^a^	60.25 ± 0.97 ^ab^	58.85 ± 0.56 ^b^	*0.0003*
Fat thickness (mm)	12.31 ± 0.29 ^b^	12.04 ± 0.30 ^b^	12.61 ± 0.52 ^ab^	13.28 ± 0.29 ^a^	*0.0005*
Muscle thickness (mm)	67.20 ± 0.61 ^a^	64.06 ± 0.61 ^c^	64.91 ± 0.79 ^bc^	65.87 ± 0.62 ^b^	*0.0008*
Belly lean meat content (%)	60.84 ± 0.33	59.82 ± 0.33	59.55 ± 0.56	59.87 ± 0.34	*0.0813*
Loin with bones (kg)	11.89 ± 0.07	11.87 ± 0.07	11.84 ± 0.11	11.69 ± 0.07	*0.0566*
Lean and boneless loin (kg)	7.52 ± 0.06	7.39 ± 0.06	7.37 ± 0.10	7.35 ± 0.06	*0.1229*
Ham with bones (kg)	24.31 ± 0.07 ^a^	23.91 ± 0.07 ^c^	23.98 ± 0.10 ^bc^	24.13 ± 0.07 ^b^	*0.0021*
Lean and boneless ham (kg)	19.00 ± 0.12 ^a^	18.60 ± 0.13 ^b^	18.60 ± 0.21 ^b^	18.60 ± 0.13 ^b^	*0.0007*
Shoulder with bones (kg)	11.86 ± 0.02 ^a^	11.81 ± 0.02 ^b^	11.81 ± 0.04 ^ab^	11.79 ± 0.02 ^b^	*0.0179*
Lean and boneless shoulder (kg)	9.23 ± 0.04 ^a^	9.18 ± 0.04 ^a^	9.15 ± 0.08 ^ab^	9.09 ± 0.04 ^b^	*0.0067*
Belly (kg)	12.95 ± 0.08	13.16 ± 0.08	13.18 ± 0.12	13.12 ± 0.08	*0.0868*

Parameters within a row with at least one identical letter are non-significant from each other (*p* < 0.05). The *p*-value (level of significance) is presented in italic letters.

**Table 3:** Proportion of carcasses (%) within one grade according to the German grading system (SEUROP) for pig carcasses of gilts, boars, immunocastrates and barrows. Distributions vary significantly from each other (*p* < 0.0001).

	Sex Group			
Grade	G (*n* = 15,213)	B (*n* = 16,903)	IC (*n* = 2812)	BA (*n* = 1976)
S	85.3%	89.3%	86.5%	47.9%
E	14.3%	10.5%	13.0%	42.1%
U	0.4%	0.2%	0.5%	9.0%
R	0.0%	0.0%	0.0%	1.0%

**Table 4:** Carcass characteristics and estimated primal pork cuts using AutoFOM III data for farms (best linear unbiased estimator (BLUP) ± SE).

Parameter	Farm										
	A	B	C	D	E	F	G	H	I	J	K
Hot carcass weight (kg)	95.15 (1.01)	93.37 (0.96)	96.24 (0.96)	94.64 (1.06)	94.11 (1.06)	94.37 (1.25)	95.42 (1.01)	94.36 (0.95)	94.37 (1.25)	92.55 (0.97)	93.52 (0.93)
Lean meat content (%)	59.17 (0.93)	62.04 (0.90)	60.36 (0.90)	60.20 (0.97)	60.32 (0.97)	60.26 (1.12)	60.55 (0.93)	58.53 (0.89)	60.26 (1.12)	61.06 (0.89)	60.10 (0.86)
Fat thickness (mm)	13.26 (0.49)	11.74 (0.47)	12.51 (0.47)	12.59 (0.50)	12.53 (0.50)	12.56 (0.57)	12.23 (0.49)	13.33 (0.47)	12.56 (0.57)	12.19 (0.46)	12.66 (0.45)
Muscle thickness (mm)	65.06 (0.88)	67.02 (0.81)	65.63 (0.82)	65.55 (0.98)	65.47 (0.98)	65.51 (1.25)	63.82 (0.88)	63.95 (0.81)	65.51 (1.25)	67.65 (0.88)	65.44 (0.82)
Belly lean meat content (%)	59.41 (0.54)	60.93 (0.51)	60.10 (0.52)	60.01 (0.55)	60.03 (0.55)	60.02 (0.63)	59.99 (0.54)	59.09 (0.51)	60.02 (0.63)	60.67 (0.52)	59.95 (0.50)
Lean and boneless loin (kg)	7.31 (0.10)	7.60 (0.09)	7.43 (0.09)	7.41 (0.10)	7.41 (0.10)	7.41 (0.12)	7.35 (0.10)	7.22 (0.09)	7.41 (0.12)	7.56 (0.10)	7.39 (0.09)
Loin with bones (kg)	11.72 (0.10)	12.03 (0.10)	11.83 (0.10)	11.82 (0.11)	11.83 (0.11)	11.82 (0.13)	11.79 (0.10)	11.63 (0.10)	11.82 (0.13)	11.96 (0.10)	11.80 (0.10)
Lean and boneless ham (kg)	18.47 (0.21)	19.10 (0.20)	18.72 (0.20)	18.70 (0.22)	18.70 (0.22)	18.70 (0.26)	18.65 (0.21)	18.30 (0.20)	18.70 (0.26)	18.99 (0.20)	18.68 (0.19)
Ham with bones (kg)	23.98 (0.11)	24.27 (0.10)	24.10 (0.10)	24.10 (0.12)	24.07 (0.12)	24.08 (0.14)	23.97 (0.11)	23.88 (0.10)	24.08 (0.14)	24.30 (0.11)	24.08 (0.10)
Lean and boneless shoulder (kg)	9.09 (0.07)	9.30 (0.07)	9.16 (0.07)	9.16 (0.07)	9.16 (0.07)	9.16 (0.08)	9.20 (0.07)	9.04 (0.07)	9.16 (0.08)	9.20 (0.07)	9.163 (0.07)
Shoulder with bones (kg)	11.78 (0.04)	11.86 (0.04)	11.82 (0.04)	11.82 (0.04)	11.81 (0.04)	11.82 (0.04)	11.83 (0.04)	11.77 (0.04)	11.82 (0.04)	11.83 (0.03)	11.81 (0.03)
Belly (kg)	13.21 (0.12)	12.84 (0.12)	13.12 (0.12)	13.10 (0.13)	13.11 (0.13)	13.10 (0.16)	13.18 (0.12)	13.34 (0.11)	13.10 (0.16)	12.93 (0.12)	13.12 (0.11)
Amount of pork per carcass (kg)	48.08 (0.26)	48.83 (0.25)	48.42 (0.25)	48.37 (0.27)	48.38 (0.27)	48.37 (0.31)	48.37 (0.26)	47.90 (0.24)	48.37 (0.31)	48.68 (0.25)	48.35 (0.24)
